# Catheter ablation of right ventricular outflow tract tachycardia using contact force guidance

**DOI:** 10.1007/s12471-013-0492-x

**Published:** 2014-01-08

**Authors:** S. D. A. Valk, N. M. S. de Groot, L. Jordaens

**Affiliations:** Department of Clinical Electrophysiology, Erasmus Medical Center, ‘s Gravendijkwal 230 room Ba 593, 3015 CE Rotterdam, the Netherlands

Outflow tract tachycardias (OTT) originating from the right or left ventricular outflow tract are thought to be benign. However, symptoms can be disabling and deterioration of left ventricular function may occur with a high arrhythmia burden. Catheter ablation has a high acute success rate of up to 90 %, but recurrences are not uncommon [1, 2]. Novel catheter designs, and mapping and ablation technologies aim to improve procedural outcome, lower the complication rate, and increase the long-term success rate [3]. The TactiCath® catheter (TactiCath®, Endosense, SA Meyrin/Geneva, Switzerland, distributed by Biotronik, Berlin, Germany) is a contact force (CF) sensing radiofrequency ablation catheter that provides real-time assessment of tip-to-tissue CF during ablation [4]. Until now, it has only been used in ablation of supraventricular tachycardias, mainly atrial fibrillation. In this report, we describe the case of a patient who underwent successful ablation of right ventricular OTT and ventricular premature beats (VPBs) using CF guidance. To the best of our knowledge, this is the first case ever described of OTT ablation using CF with the TactiCath® catheter.

## Case report

**Fig. 1:**
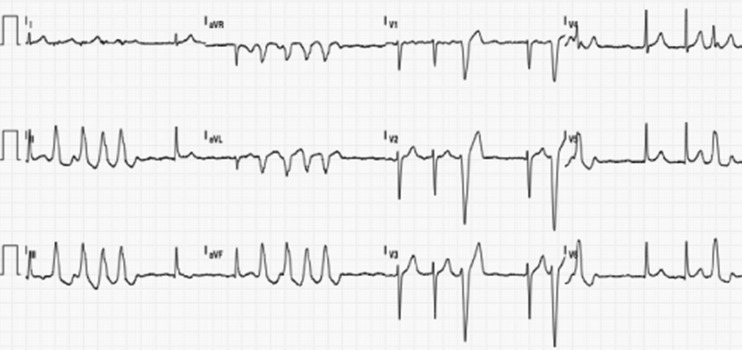
Electrocardiogram showing atrial fibrillation with nonsustained ventricular tachycardia with left bundle branch block, inferior axis and late QRS transition

**Fig. 2:**
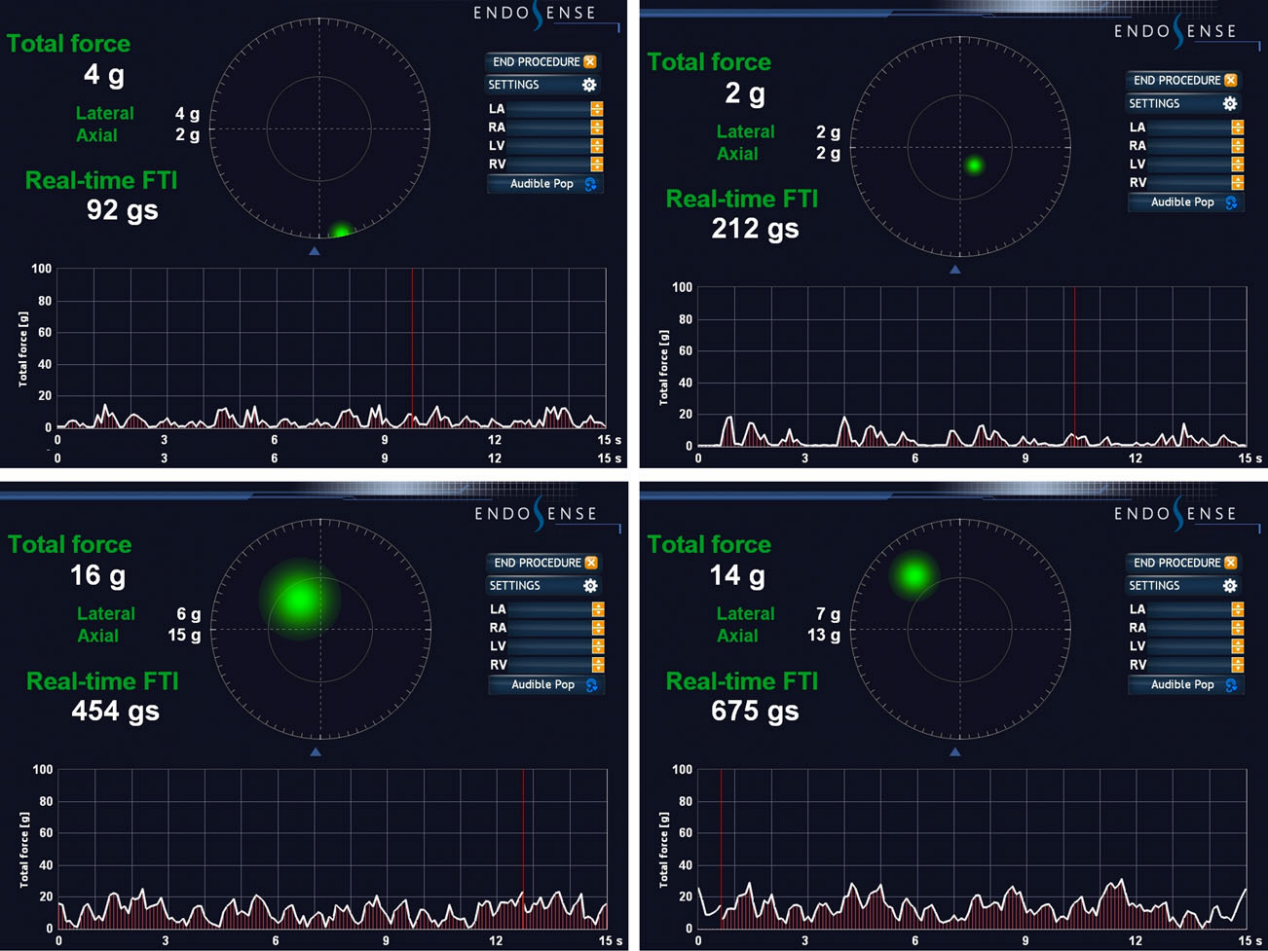
Contact force of the first (*upper half*) and second (*lower half*) application. Snapshots of the first and last 15 s at the left and right, respectively. In the first application the contact force, given for both axial and lateral contact is low. During the second application an almost constant force is developed in the axial direction, as shown by the curve versus time, and illustrated by the large central green spot

**Fig. 3:**
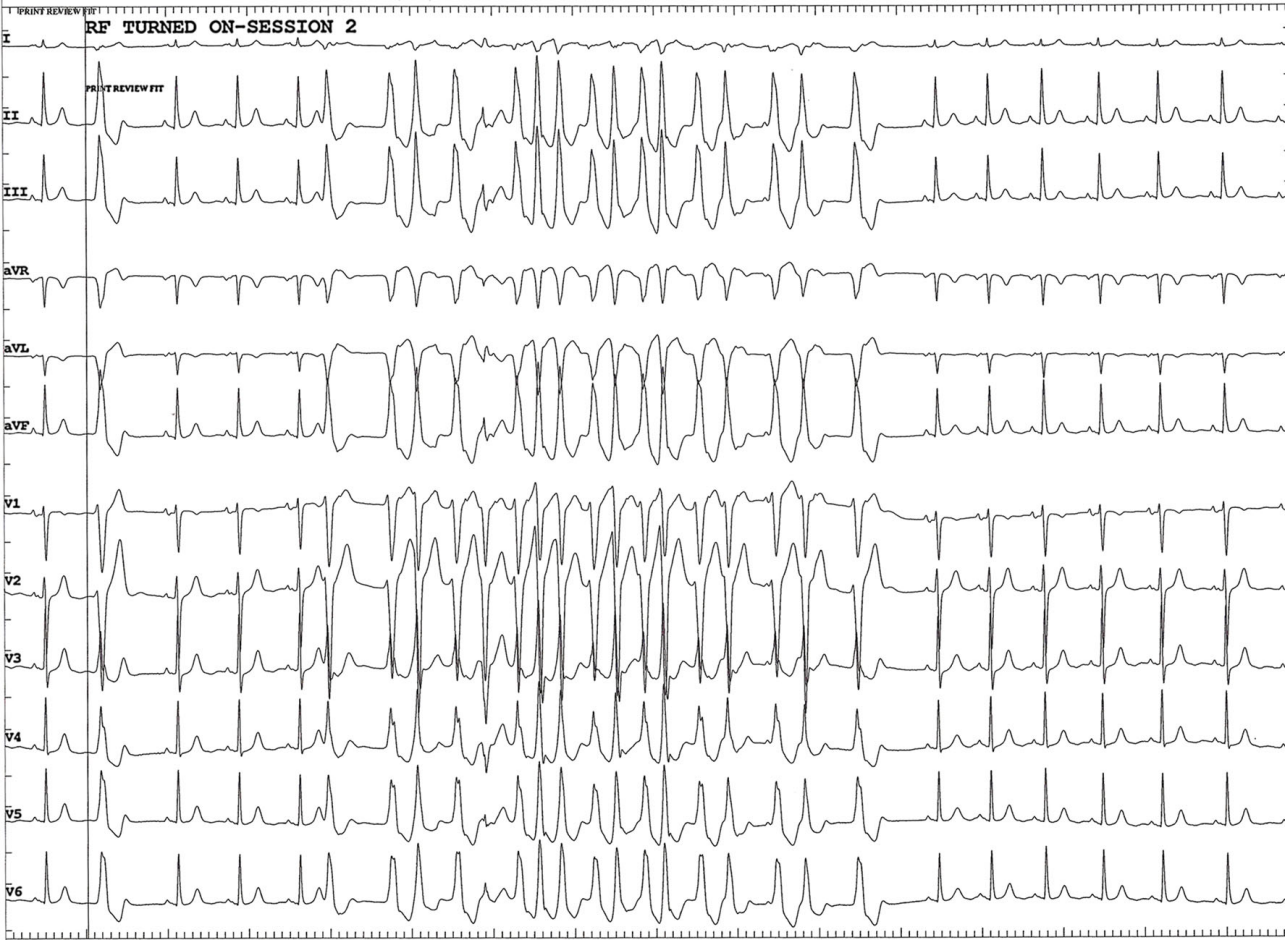
Clinical arrhythmia immediately starting up after the onset of ablation, to disappear within 10 s

A 60-year-old patient was referred to the Erasmus Medical Center for palpitations and dizziness. Electrocardiograms showed repetitive nonsustained ventricular tachycardia (VT). She developed sustained VT during exercise testing which terminated spontaneously after cessation of the test. The arrhythmia had left bundle branch morphology, an inferior axis, and QRS transition zone in lead V4, suggestive of a right ventricular outflow tract (RVOT) origin (Fig. 1). Structural cardiac disease was excluded. Electro-anatomical mapping (NaVX, Endocardial Solutions, St. Jude Medical, Inc., St. Paul, MN, USA) was used to create an anatomical reconstruction of the RVOT. Activation mapping during VPBs revealed the earliest site of activation in the low anteroseptal area of the RVOT. Pacing at this site resulted in a 12/12 pacemap, and the TactiCath® catheter was used to deliver two applications of 60 s guided by CF sensing (Fig. 2). Although the CF during the first application was applied axially, it resulted in a low force-time integral (calculation of force over time) and the application was not successful. The second application was delivered at the same site as the first. It started with 15 g of axial CF, which induced and then terminated the clinical VT during ablation (Fig. 3). The application was delivered with the same force, ending with a force time integral of 675 gram-seconds. After the second application, the patient was free of both VT and VPBs.

## Discussion

The recurrence rate of outflow tract arrhythmias after catheter ablation is high [1, 2]. This may be due to resolving of initial tissue oedema, which may have mimicked an initial transmural, effective lesion. It may also be caused by difficulties in obtaining and retaining a stable catheter position with adequate tissue contact during ablation. The TactiCath® catheter was developed to measure CF between the tip of the ablation catheter and cardiac tissue. It is an open-irrigation catheter, containing a triaxial force sensor located between the second and third electrode that measures amplitude and direction of the force applied between the tissue and electrode tip. The sensor has a resolution and sensitivity of 1 g in a bench test [4]. The electrophysiologist is continuously, real-time, informed if the CF is changing, which is a sign of too much or too little tissue contact, and adjustments in CF can be made instantaneously. Adequate and constant tissue contact may reduce the number of applications with inadequate tissue contact, which may potentially lead to insufficient lesion formation and hence arrhythmia recurrences. This technique may increase patient safety as excessive CF on the myocardium is avoided (less risk for perforation and thrombus formation). This may also lead to shorter procedure times, with less radiation exposure for both patient and the operator. Previously, this new technique was only employed in supraventricular tachycardia (atrial fibrillation) ablation, but this case shows that it may also be feasible in ablation of outflow tract arrhythmias. To the best of our knowledge, this is the first report on contact force technology used for ablation in the right ventricular outflow tract. The patient was treated successfully with one effective application after a previous application with inadequate CF. She did not experience any complications. This technique, therefore, seems very promising. In the future, indications for the use of CF-guided ablation may further expand and may include children in which less radiation exposure is even more important [5]. Ideally, CF technology could be integrated in magnetic ablation catheters to make magnetic navigation procedures even more safe and precise, with potentially an even lower complication rate compared with the already low complication rate associated with magnetic navigation procedures [6]. Further studies are needed to evaluate the use of CF in OTT ablation, and to develop a range of optimal CF in this area to create a transmural lesion without perforating the myocardium.
